# Partial Clinical Remission of Type 1 Diabetes: The Need for an Integrated Functional Definition Based on Insulin-Dose Adjusted A1c and Insulin Sensitivity Score

**DOI:** 10.3389/fendo.2022.884219

**Published:** 2022-05-03

**Authors:** Benjamin Udoka Nwosu

**Affiliations:** Division of Endocrinology, Department of Pediatrics, Zucker School of Medicine at Hofstra/Northwell, New Hyde Park, NY, United States

**Keywords:** type 1 diabetes, Honeymoon phase, partial clinical remission, insulin dose adjusted A1c, insulin sensitivity

## Abstract

Despite advances in the characterization of partial clinical remission (PR) of type 1 diabetes, an accurate definition of PR remains problematic. Two recent studies in children with new-onset T1D demonstrated serious limitations of the present gold standard definition of PR, a stimulated C-peptide (SCP) concentration of >300 pmol/L. The first study employed the concept of insulin sensitivity score (ISS) to show that 55% of subjects with new-onset T1D and a detectable SCP level of >300 pmol/L had low insulin sensitivity (IS) and thus might not be in remission when assessed by insulin-dose adjusted A1c (IDAA1c), an acceptable clinical marker of PR. The second study, a randomized controlled trial of vitamin D (ergocalciferol) administration in children and adolescents with new-onset T1D, demonstrated no significant difference in SCP between the ergocalciferol and placebo groups, but showed a significant blunting of the temporal trend in both A1c and IDAA1c in the ergocalciferol group. These two recent studies indicate the poor specificity and sensitivity of SCP to adequately characterize PR and thus call for a re-examination of current approaches to the definition of PR. They demonstrate the limited sensitivity of SCP, a static biochemical test, to detect the complex physiological changes that occur during PR such as changes in insulin sensitivity, insulin requirements, body weight, and physical activity. These shortcomings call for a broader definition of PR using a combination of functional markers such as IDAA1c and ISS to provide a valid assessment of PR that reaches beyond the static changes in SCP alone.

## LITERATURE SEARCH CRITERIA

A literature search was conducted to identify publications addressing the definitions of partial clinical remission (PR) in children and adults. Medline, EMBASE, and Ovid were searched using the following search terms: clinical remission, partial remission, partial clinical remission, honeymoon phase, C-peptide, type 1 diabetes, children, pediatric type 1 diabetes, and paediatric type 1 diabetes.

## Introduction

The definition of partial clinical remission (PR) remains problematic (1). Type 1 diabetes (T1D) is associated with reduced insulin sensitivity (IS) (2), but the adoption of the measurement of serum C-peptide (SCP) concentration as a primary endpoint in clinical studies on PR (3–7) does not accurately capture this reduced IS state (8). Furthermore, SCP has several physiological, biochemical, and pharmacokinetic limitations that affect its specificity and sensitivity (9, 10). Despite these shortcomings, the American Diabetes Association recommends SCP as the gold standard test for the assessment of PR in clinical trials (11). In contrast, the International Society for Pediatric and Adolescent Diabetes (ISPAD) recommends the insulin-dose adjusted A1c (IDAA1c) as its gold standard test for PR (12, 13). However, both SCP and IDAA1c do not directly assess insulin resistance or sensitivity (1) which limits the ability of each formula to fully characterize the heterogeneity of T1D and its multiple endotypes (14).

Diabetes mellitus affects 34.2 million, or 10.5% of the US population (15). T1D is a syndrome of persistent hyperglycemia secondary to insulinopenia resulting from autoimmune destruction of pancreatic β-cells (16). At the time of diagnosis of T1D, approximately 50% of residual β-cell function (RBCF) may remain, and this RBCF may persist for months or years (17–19). Prolonging the partial clinical remission (PR), also known as the ‘honeymoon’ phase of T1D improves glycemic control and reduces long-term complications (1, 20). Interventions to prevent immune-mediated destruction of β-cells with immunosuppressive and immunomodulatory agents have yielded promising trends, but insufficient protection (3–6). The primary outcome in these interventions is the SCP level (21). However, T1D is a heterogeneous disease with multiple endotypes (14), and recent data on the shortcomings of SCP as a primary endpoint in clinical studies on PR suggest that the negative results from some prior trials may be due to the limitations of SCP as a marker of PR, and not necessarily on the shortcomings of the study designs. Recent research suggests that functional parameters could provide a more robust characterization of PR(1, 13) (12) in a manner similar to the superiority of the measurement of plasma renin activity, a dynamic test, to plasma renin, a static test, in the assessment of the renin-angiotensin system (22).

## Newer Discoveries in the Field: A Role for the Synergistic Impact of Insulin-Sensitivity Scores (ISS) and Insulin-Dose Adjusted A1c

Despite its approval by the American Diabetes Association (11), there is accumulating evidence in the literature that the current gold standard definition of PR, the SCP, is imprecise and underestimates PR, and in most cases fails to detect PR when present (8, 19, 23). Two recent studies suggest that the use of a combination of IDAA1c and ISS to confirm PR is more precise and specific (8, 23). Clinically, PR is defined by IDAA1c, a two-dimensional marker of PR that correlates insulin dose and measured HbA1c to residual β-cell function (24). IDAA1c has good agreement with stimulated C-peptide level of >300 pmol/L (25) which is currently considered the gold standard definition of PR, but IDAA1c has more utilitarian value as it also reflects changes in insulin requirements, body weight, and conditions that impact insulin requirements such as exercise. The IDAA1c is calculated by:


HbA1c (%)+[4x insulin dose(units/kg/24h)]

where PR is defined as IDAA1c of ≤9 (24).

In a recent pivotal longitudinal study, Mork et al (8) used the formula for insulin sensitivity score (ISS) developed by Dabelea et al (26) to determine the differences in ISS among youth in PR as defined by stimulated C-peptide level of >300 pmol/L (>2 ng/mL). The formula is:


$$\text{lo}{\text{g}}_{\text{e}}\text{IS}=4.64725–0.02032\left(\mathrm{waist},\;\mathrm{cm}\right)–0.09779\;(\text{Hb}{\text{A}}_{1\text{c}},\%-0.00235\;(\text{TG},\;\text{mg}g/\text{dL}$$

[to convert triglycerides (TG) values from mmol/L to mg/dL, divide by 0.0113)] (26). Using the formula to generate ISS for each subject, Mork reported that 55% of their participants with detectable SCP of >300 pmol/L had low ISS and thus might not be in PR when defined by a functional marker of PR, the IDAA1c, suggesting that the presence of detectable SCP of >300 pmol/L (>2 ng/mL) does not equate to PR. Therefore, isolated use of SCP could have overestimated the number of subjects in PR in their cohort.

**Figure 1:**
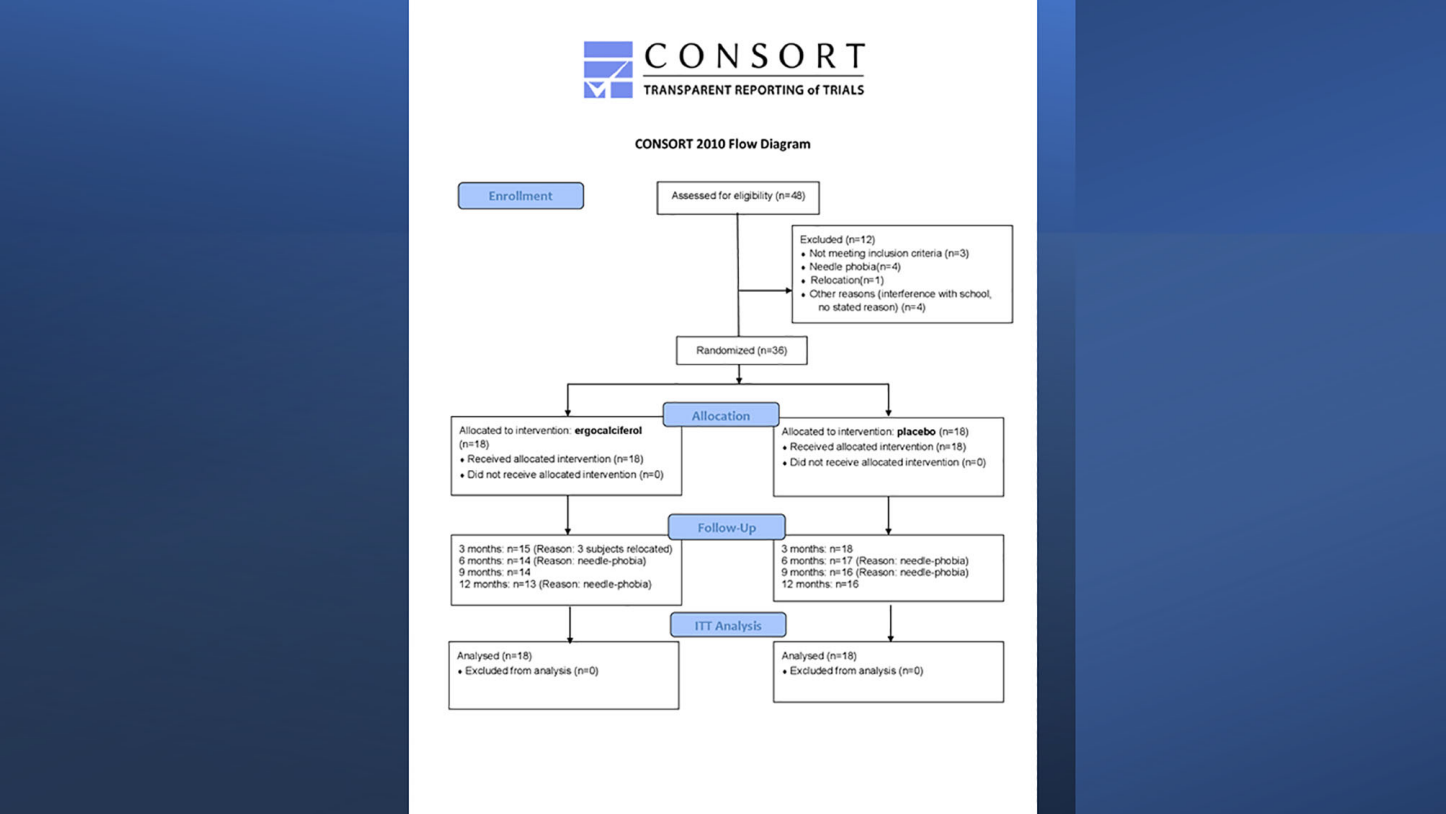
Consort Flow Diagram of randomized controlled trial of ergocalciferol in children and adolescents with newly diagnosed type 1 diabetes.

The discordance between SCP and PR was recently characterized by Nwosu et al (23) who randomized 36 subjects of 10-21 years with newly-diagnosed T1D in a 12-month randomized controlled trial (RCT) of ergocalciferol versus placebo to determine the impact of vitamin D on residual β-cell function (RBCF) and PR in youth with newly-diagnosed T1D **(** **Figure 1** **)**. The trial’s hypothesis was that adjunctive ergocalciferol would increase RBCF and prolong PR. The primary aim was to determine the effect of ergocalciferol on RBCF and PR in children and adolescents with T1D, while the primary outcome was the longitudinal change in RBCF as marked by SCP and IDAA1c.

**Figure 2:**
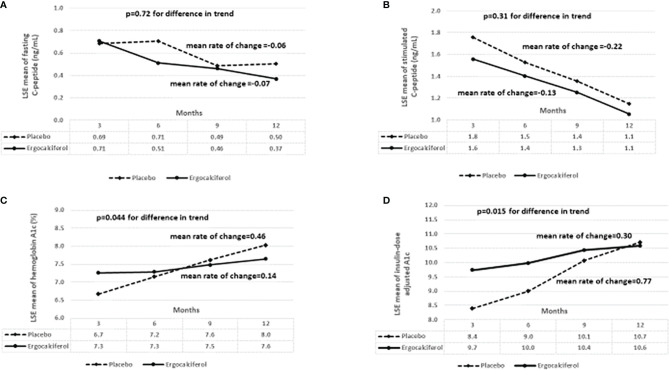
**(A)** shows the trend analysis of the least square estimates (LSE) of the means for fasting C-peptide. There was no significant difference in fasting C-peptide concentration between the ergocalciferol and placebo groups during the 12-month trial, (p=0.72). **(B)** There was no significant difference in the change in stimulated C-peptide concentration at 90 minutes between the ergocalciferol- and placebo-treated patients with type 1 diabetes during the 12-month trial, (p=0.31). **(C)** Temporal trend analysis showed a faster rate of rise in hemoglobin A1c (HbA1c) values in the placebo group compared to the vitamin group (p=0.044). **(D)** Temporal trend analysis showed a significantly faster rise in insulin dose adjusted A1c (IDAA1c) in the placebo group compared to the ergocalciferol group (p=0.015). This suggests beta-cell protection by ergocalciferol.

There were no differences in basal and SCP between the groups for the duration of the trial **(** **Figures 2A, B** **)**. In contrast, there was a significant blunting of the temporal rise in both IDAA1c and A1c in the ergocalciferol group compared to the placebo group **(** **Figures 2C, D** **)**. Specifically, the placebo group showed a faster rate of increase in HbA1c at a mean rate of change of 0.46% every 3 months, compared to the slower rate of change in the ergocalciferol group, mean rate of change of 0.14% every 3 months, (p=0.044). There was equally a faster rate of rise in IDAA1c in the placebo group at a mean rate of change of 0.77 every 3 months, whereas the rate of rise in the ergocalciferol group was significantly blunted, at a mean rate of change of 0.30 every 3 months (p=0.015).

In contrast to the study by Mork et al, a 2016 observational study of 407 patients of 3-45 years by Hao et al (7) recommended that investigators use SCP as the primary endpoint in PR studies because IDAA1c significantly underestimated the number of subjects with SCP of ≥0.2 nmol/L, especially in children. This provided support for the 2004 decision by the ADA to adopt SCP as the gold-standard marker for assessing PR in clinical studies (11). However, in a landmark 2019 study of 1019 individuals with T1D for more than 50 years, who were prospectively followed for 4 years, Yu et al (19) reported that only 32% of the subjects had documented measurable SCP after using a combination of MMTT and hyperglycemic clamp with arginine infusion to estimate SCP; even though all 68 studied pancreases of their cohort were positive for insulin-containing β-cells. This discordance in premortem SCP and postmortem pancreatic histology, despite rigorous evaluation of pancreatic C-peptide secretory ability, led the authors to conclude that residual β-cells were present in individuals with T1D regardless of disease duration or measurable SCP levels. Interestingly, the average A1c for this cohort was 7.1%, suggesting that if the investigators had calculated the participants’ IDAA1c values, the mean value would have been mostly ≤9, suggesting that IDAA1c is more sensitive than SCP for PR detection. This is in line with the decision by ISPAD to designate IDAA1c as the gold-standard marker for PR (12).

A recent review article by Oram et al (27) reported a wide gap in the range of SCP positivity of 11% to 80% despite the fact that these studies used C-peptide assays of improved sensitivity and specificity in heterogenous groups of patients with long-standing T1D (18, 28–31). The detected C-peptide levels from these studies were mostly at very low concentrations. For example, the study by Wang et al (29), which used an ultrasensitive C-peptide assay, only detected SCP in 10% of the subjects, while the T1D Exchange study by Davis et al (30) detected only 29% SCP positivity using both basal and stimulated C-peptide measurements. The authors suggested that variations in SCP in these studies most likely resulted from cohort selection criteria, cohort ages and duration of T1D, differing assay sensitivities, and sample storage conditions. This wide discrepancy in SCP detection in various studies, and the range of factors that undergird its detectable concentration, limit its usefulness as a primary marker of PR as detailed below.

## Limitations of Serum C-Peptide as a Primary Marker of Partial Clinical Remission of Type 1 Diabetes

The gold standard definition of PR is the detection of a stimulated C-peptide level (SCP) of >300 pmol/L following a mixed meal tolerance test (23, 32). Recent publications indicate that this one-dimensional definition may be insufficient to adequately address the components of a complex, multifaceted process such as PR[1, 13 (14)]. SCP is a biochemical marker with several limitations that diminish its use as a gold standard criterion for PR (27). Some of these limitations are physiological, anatomical, biochemical, pharmacokinetic, and glycemic related as detailed below.

### Physiological Limitations

The major detriment to the use of SCP concentration to confirm PR is that its peak level depends on several physiological processes which may be deranged in patients with T1D. The are 2 primary provocative tests for SCP: the less popular glucagon stimulation test (GST) directly stimulates the β-cells to release C-peptide (33) while the more popular test, the mixed meal tolerance test (MMTT) stimulates insulin secretion from β-cells following the ingestion of a meal consisting of proteins, fats, and carbohydrates (34). A recent study (32) in healthy subjects without diabetes mellitus that compared GST and MMTT found that while GST directly stimulates the β-cells to release C-peptide, MMTT relies on an intact incretin axis that consists of operative gastric inhibitory peptide (GIP) and glucagon-like peptide-1 (GLP-1) systems. However, the responsiveness of the GIP/GLP-1 system is reduced in patients with recent onset T1D (9). Thus, the results of MMTT depends on the robustness and the combined action of the GIP and GLP-1 (34), and the type of meal used for the study (35). The reduced responsiveness of the GIP/GLP-1 systems in patients with recently diagnosed T1D suggests that the use of SCP as the primary outcome in these patients may underestimate their residual β-cell function (23). However, no study has investigated the impact of an altered incretin axis on β-cell response during MMTT (32) to validate the results obtained in trials relying on MMTT for SCP, as well as the adjustments needed for peak SCP concentrations in situations of suspected poor incretin response. This will prevent false negative results, and false negative conclusions in clinical trials.

The physiological significance of different concentrations of peak SCP following MMTT were recently examined by Rickels et al (36) who studied 63 adult patients who were stratified into 4 groups by their peak SCP concentration from MMTT. Using both hyperinsulinemic euglycemic clamp technique and hyperinsulinemic hypoglycemic clamping, the investigators showed that the group with the highest peak C-peptide concentration of >0.4 pmol/mL demonstrated robust β-cell responsiveness to hyperglycemia, as well as a robust α-cell responsiveness to hypoglycemia. This physiological adaption was absent in the groups with either negative or low peak C-peptide concentration. This α and β cell responsiveness to high peak C-peptide levels in patients with T1D may explain the significantly better glycemic control in remitters versus nonremitters. Thus, the transition from preclinical to clinical T1D is marked by progressive loss of β-cells with attendant inability of β-cells to suppress glucagon-mediated hyperglycemia. This study showed that residual C-peptide concentration of >0.4 pmol/mL following MMTT is a threshold of physiologic importance for both α-cell responsiveness to hypoglycemia and β-cell responsiveness to hyperglycemia. However, this responsiveness to hyperglycemia is incomplete as no amount of residual C-peptide can fully suppress glucagon once a diagnosis of T1D is made. Furthermore, sub-threshold residual C-peptide concentrations appear to be physiologically inactive.

### Anatomical Limitations

T1D is a heterogenous disease with multiple endotypes (14). Recent reports of proinsulin production and secretion in most patients with long-standing T1D, including those without measurable C-peptide, suggest the presence of anatomic defect at the tissue level (27). This defect is associated with markedly reduced insulin production, abnormal hormone processing, with attendant proinsulin accumulation in the pancreas and general circulation (37, 38). This phenomenon suggests the existence of β-cells that can start the process of hormone production but are incapable of releasing mature insulin and C-peptide (27). Interestingly, proinsulin to C-peptide ratio, a marker of β-cells endoplasmic reticulum stress, is increased in children and adolescents with new-onset T1D (39). Thus, this could represent a significant limitation to the use of serum C-peptide to characterize PR in children. The existence of this subpopulation of β-cells is supported by recent reports that T1D is a heterogenous disease (14) marked by histopathological heterogeneity (40). Thus, a simple static test such as SCP may not adequately reflect the anatomic heterogeneity of the surviving β-cells.

### Biochemical Limitations

The biochemical limitation of SCP assay were reported in a study (10) that examined the stability of C-peptide among 6 different assays. This study concluded that C-peptide stability varies significantly with different assay methods, and the stability for all assays is < 1 month, even when LC/MS/MS is used (41). It further reported that C-peptide is more stable in plasma than in serum, and that adding aprotinin to serum or plasma does not significantly improve the stability of C-peptide for any of the methods. Additionally, serum C-peptide degrades easily on storage (42) such that batched samples could easily give spuriously low readings leading to false negative results of SCP assay, which otherwise could be easily verified as inaccurate by the inclusion of the functional markers, IDAA1c and ISS.

Oram et al (27) reported that even under standardized protocols for pre-analytic handling of samples, the results of SCP could show wide inter-study variability. For example, whereas Mork (8), Rickels (36), and Mortensen (25) showed adequate C-peptide response in their observational studies, the RCT by Nwosu et al (23) did not detect any significant difference in SCP between the placebo and the experimental groups despite the detection of significantly lower temporal trends in the rise in both IDAA1c and A1c in the experimental group. Additionally, Yu et al (19) in their landmark study found no correlation in SCP detection by MMTT and hyperglycemic clamp with arginine infusion. In that study, the lack of correlation between premortem SCP levels and postmortem pancreatic morphological examination in the same patients in their cohort was so discrepant that the investigators concluded that regardless of measurable C-peptide levels or disease duration, residual β-cells were present in individuals with T1D.

### Pharmacokinetic Properties

Earlier studies had highlighted the controversies surrounding the use of C-peptide to quantify insulin secretion because the kinetics of C-peptide under different conditions are not clearly defined (43). C-peptide is co-secreted with insulin and its serum concentration is dependent on the rate of production, volume of distribution, and renal clearance (44, 45). Therefore, factors that impair its production, volume of distribution, and clearance could easily lead to inaccurate results.

### Glycemic and Related Limitations

Finally, SCP on its own is unable to detect changes in insulin resistance (IR), insulin sensitivity (IS) (8), insulin requirements, or changes in body weight. This contrasts with the functional markers, IDAA1c and ISS whose values change with conditions that alter insulin sensitivity and body weight. Though IDAA1c does not directly measure IS, the formular for ISS assesses IS directly.

## Implications for Studies in the Field of Partial Clinical Remission

There are several implications for studies in the field. The first is the recognition that T1D is a heterogenous, complex disease with several endotypes, and over-reliance on SCP, a marker with numerous limitations, as the gold-standard definition of PR that could easily lead to negative study results in situations where functional definitions of PR could easily show a positive signal or result. Isolated use of SCP could easily under-estimate the proportion of subjects undergoing PR, a scenario that would be easily clarified using a combination of functional markers. The over-reliance on SCP fails to address the differences in IS status that is intrinsic to T1D and thus could lead to an over-estimation of PR in a population. In light of conclusions from recent studies (1, 13) (14), the overreliance on SCP as a study endpoint may be too simplistic and may not provide the full picture of the changes in insulin requirements, IS, and body weight, which are captured by the functional markers. SCP could vary by assay technique and thus may be subject to error. Serum C-peptide degrades easily and thus needs to be run within one week of collection. Therefore, batched study samples could easily lead to spuriously low results and under-estimation of PR.

## Conclusions and Recommendations

This review recognizes that T1D is a heterogenous disease with multiple endotypes and recommends that studies on PR should use a combination of IDAA1c and ISS to first establish PR, and the changes in IS during PR to provide a comprehensive picture of the pathophysiology of PR. Using this approach, SCP will serve as a secondary marker of PR. This paradigm will enable future clinical trials on PR to employ multi-faceted approaches to the definition of PR and not rely solely on SCP. Therefore, clinical trials will focus more on the impact of interventions on functional parameters such as IDAA1c and IS, and secondarily on SCP. This will help obviate the imprecise results of clinical trials arising from the shortcomings of SCP.

## Future Directions

A change in the characterization of PR with emphasis on functional markers as gold standard tests might reveal that some of the earlier studies that showed no significant difference in SCP between the treatment and placebo arms might demonstrate positive results when assessed using functional markers. For example, some of the studies examining the impact of immunomodulatory agents on PR could use a combination of IDAA1c and ISS as their endpoints. This change in the definition of PR will improve sensitivity and specificity and accelerate the pace of research in the field of partial clinical remission and its clinical applications. This will move the field forward.

## Author Contributions

The author confirms being the sole contributor of this work and has approved it for publication.

## Funding

This study was funded in part by an investigator-initiated research grant, Grant ID: 1 R21 DK113353-03, to BN from NIDDK, NIH.

## Conflict of Interest

The author declares that the research was conducted in the absence of any commercial or financial relationships that could be construed as a potential conflict of interest.

## Publisher’s Note

All claims expressed in this article are solely those of the authors and do not necessarily represent those of their affiliated organizations, or those of the publisher, the editors and the reviewers. Any product that may be evaluated in this article, or claim that may be made by its manufacturer, is not guaranteed or endorsed by the publisher.
